# A preliminary study developing a scoring model incorporating fibrinogen-like protein 2 for predicting glucocorticoid resistance in thyroid eye disease

**DOI:** 10.1186/s13044-026-00293-8

**Published:** 2026-04-01

**Authors:** Kazuhiko Matsuzawa, Shoichiro Izawa, Kanako Kadowaki, Kenji Fukaya, Kazuhisa Matsumoto, Keiko Nagata, Tsuyoshi Okura, Shinya Fujii, Dai Miyazaki, Shin-Ichi Taniguchi, Kazuhiro Yamamoto, Takeshi Imamura

**Affiliations:** 1 Division of Pharmacology, Tottori University Faculty of Medicine, Yonago, Tottori Japan; 2 Division of Cardiovascular Medicine, Endocrinology and Metabolism, Tottori University Faculty of Medicine, Yonago, Japan; 3 Division of Radiology, Department of Multidisciplinary Internal Medicine, Tottori University Faculty of Medicine, Yonago, Japan; 4 Division of Ophthalmology and Visual Science, Tottori University Faculty of Medicine, Yonago, Japan; 5 Department of Community-Based Family Medicine, Faculty of Medicine, Tottori University, Yonago, Japan; 6 National Cerebral and Cardiovascular Center, Osaka, Japan

**Keywords:** Thyroid eye disease, Intravenous glucocorticoid, FGL2, Biomarker, Predictive model

## Abstract

**Objective:**

Intravenous glucocorticoids are the first-line therapy for moderate-to-severe active thyroid eye disease (TED), but some patients demonstrate steroid resistance. Therefore, identifying reliable pre-treatment biomarkers to predict the therapeutic response to intravenous glucocorticoids is of considerable clinical importance.

**Methods:**

In the discovery phase, pre-treatment serum samples from 10 patients with TED treated with intravenous glucocorticoids (six responders, four non-responders), along with samples from three patients with Graves’ disease without TED and three healthy controls, were subjected to data-independent acquisition proteomics analysis. Candidate biomarkers were validated in an expanded cohort (21 responders, 10 non-responders, 14 Graves’ disease patients, and 14 healthy controls).

**Results:**

Data-independent acquisition proteomics analysis revealed 263 differentially abundance proteins between responders and non-responders. Among them, transforming growth factor-β, heparanase, and fibrinogen-like protein 2 were identified as potential biomarker candidates. In the validation phase, only fibrinogen-like protein 2 was significantly elevated in non-responders. Although fibrinogen-like protein 2 alone achieved an area under the curve of 0.76, Least Absolute Shrinkage and Selection Operator regression incorporating clinical parameters identified smoking and high thyroid-stimulating antibody as additional predictors. A three-factor scoring system (fibrinogen-like protein 2 > 39.5 ng/mL, thyroid-stimulating antibody > 2597%, and current smoking; each 1 point) yielded an area under the curve of 0.86, with good reproducibility in bootstrap validation.

**Conclusions:**

Elevated pre-treatment serum fibrinogen-like protein 2 is a promising biomarker for predicting steroid resistance in TED. Combining fibrinogen-like protein 2 with thyroid-stimulating antibody and current smoking provides a clinically useful scoring system to guide personalized treatment.

**Supplementary Information:**

The online version contains supplementary material available at 10.1186/s13044-026-00293-8.

## Introduction

Thyroid eye disease (TED) is an orbital inflammatory disorder associated with autoimmune thyroid disease [1]. Its clinical course typically consists of an active inflammatory phase followed by an inactive phase [2]. Intravenous glucocorticoid (ivGC) therapy is an established treatment for TED during the active phase [3].

The Clinical Activity Score (CAS), which consists of seven clinical signs and symptoms of inflammation, is the most widely used tool for assessing TED activity [4]. In addition, high signal intensity on T2-weighted or fat-suppressed orbital magnetic resonance images suggests active inflammation [5, 6].

Despite there being these indicators of TED activity, the reported response rate to ivGC therapy in patients with TED is as low as 50%–80% [2]. Furthermore, ivGC therapy is associated with a high incidence of adverse effects, ranging from mild to life-threatening, although the latter are rare [7–9]. One possible explanation for the suboptimal response to ivGC therapy is that some patients, even during the active phase of TED, are inherently resistant to steroids. Indeed, several studies have reported that tocilizumab is effective for ivGC-refractory TED [10–11]. If steroid resistance can be predicted before treatment, alternative first-line therapies could be considered, potentially avoiding unnecessary adverse effects. However, no objective and quantitative criteria currently exist to identify patients with ivGC-refractory TED.

Data-independent acquisition (DIA) proteomics is an analysis approach that is particularly well-suited for biomarker discovery. It enables comprehensive, unbiased, and reproducible quantification of a wide range of serum proteins without prior target selection, allowing for the detection of both known and novel candidates in complex clinical samples.

In this study, we report the identification of a serum biomarker candidate associated with ivGC treatment responsiveness in patients with TED. We first conducted an exploratory DIA proteomics analysis. We then performed a validation study using an expanded patient cohort to measure the serum concentrations of the candidate proteins identified in the discovery phase by enzyme-linked immunosorbent assay (ELISA). Finally, we developed an optimal scoring system that combines other factors with the identified serum biomarker, which may help to predict the therapeutic response to ivGC therapy in patients with TED.

## Materials and methods

### Study design and participants

This study was conducted in accordance with the Declaration of Helsinki and approved by the Ethics Committee of the Faculty of Medicine, Tottori University (approval no. 24A122). It was prospectively registered in the University Hospital Medical Information Network (UMIN) Clinical Trials Registry (UMIN-CTR ID: UMIN000055754) on December 25, 2018. Written informed consent was obtained from all participants after full explanation of the purpose and nature of all procedures used. Those who declined or could not be adequately informed were excluded.

The study comprised three phases: (1) identification of candidate predictive serum biomarkers of a therapeutic response by DIA proteomics analysis; (2) validation using commercial ELISA kits; and (3) development of a scoring system incorporating the novel serum biomarker to predict the therapeutic response to ivGC therapy in patients with TED.

Participants included those with TED, Graves’ disease, and healthy controls. Eligible participants were aged ≥ 18 years who had not received systemic glucocorticoids, immunosuppressive agents, or other treatments that could modify immune activity prior to enrollment. The TED group consisted of patients with active, moderate-to-severe TED who attended Tottori University Hospital between 2019 and 2023 and who were eligible for ivGC therapy, and had no prior history of TED-directed treatment, including systemic glucocorticoids, orbital radiotherapy, or orbital surgical interventions. Active TED was defined as a CAS of ≥ 3 out of 7 according to the European Group on Graves’ Orbitopathy (EUGOGO) guidelines, and severity was classified according to the latest EUGOGO criteria [2]. Patients with TED were classified as responders or non-responders. Responders were defined as those with a CAS reduction of ≥ 2 points or a CAS of < 3 out of 7 at 24 weeks since the initiation of ivGC therapy; others were classed as non-responders [12]. Patients with Graves’ disease were defined as those with hyperthyroidism with positive thyroid-stimulating hormone (TSH) receptor antibody in the absence of clinical signs of TED. Healthy controls had normal thyroid function, were negative for thyroid antibodies, and had no ocular symptoms.

### Ophthalmological assessments

The CAS was evaluated using the EUGOGO-modified patient form, which assesses seven signs (retrobulbar pain, pain on eye movement, conjunctival hyperemia, eyelid redness, chemosis, caruncle swelling, eyelid swelling). Diplopia was graded as 0 (none), 1 (intermittent), 2 (on gaze), or 3 (constant on straight gaze) using the same form. The Hess screen test enabled objective assessment of diplopia. Proptosis was measured using a Hertel exophthalmometer, and intraocular pressure (IOP) was measured using a non-contact tonometer (TX-20, Canon, Tokyo, Japan). The CAS was recorded at diagnosis and at 6 months after the initiation of ivGC therapy, while proptosis, diplopia, and IOP were measured at diagnosis by an ophthalmologist.

### Treatment protocol

The ivGC regimen consisted of 1 g/day intravenous methylprednisolone for three consecutive days, repeated the following week. Oral prednisolone (20 mg/day) followed, which was tapered by 5 mg/month for 2 months, and then by 2.5 mg/month, and it was finally discontinued at 6 months. The ivGC regimen was a modified version of the 9 g protocol [13]. Orbital radiotherapy was administered (20 Gy over 2 weeks) to all except two patients aged < 35 years. In patients who received orbital radiotherapy, treatment was initiated concurrently with the start of ivGC in all cases.

### Blood examinations

Free thyroxine, free triiodothyronine, TSH, TSH receptor antibody, thyroid peroxidase antibody, and thyroglobulin antibody were measured by electrochemiluminescence immunoassays (Elecsys, Roche Diagnostics, Mannheim, Germany; reference ranges: 0.93–1.70 ng/dL, 2.30–4.00 pg/mL, 0.27–4.20 µIU/mL, < 2.0 IU/L, < 16 IU/mL, and < 28 IU/mL, respectively). Thyroid-stimulating antibody (TSAb) was measured using a commercial kit (Yamasa, Tokyo, Japan; cutoff 110%).

### DIA proteomics analysis

Proteomics analysis was performed by DIA mass spectrometry (MS) at BGI Japan (Kobe, Japan), following their standard experimental and bioinformatics pipelines. The DIA workflow consisted of spectral library construction using data-dependent acquisition (DDA), DIA quantification in SWATH-MS mode, and comprehensive bioinformatics analysis.

### Protein extraction and digestion

Blood samples were collected before ivGC therapy, promptly frozen at − 80 °C, and stored until analysis. For each sample, 100 µL serum was mixed with sodium dodecyl sulfate (SDS)-free lysis buffer to a final volume of 1 mL. Proteins were reduced using 10 mM dithiothreitol at 37 °C for 30 min, alkylated with 55 mM iodoacetamide at room temperature in the dark for 30 min, and enriched by solid-phase extraction using a C18 column. Protein concentration and quality were assessed by the Bradford assay and SDS-polyacrylamide gel electrophoresis. For digestion, 100 µg protein was diluted 4-fold with 50 mM ammonium bicarbonate, incubated with trypsin at a protein: enzyme ratio of 40:1 for 4 h at 37 °C, desalted using a Strata X column, and vacuum-dried.

### High pH reverse phase separation

Equal amounts of peptide from all samples were pooled, diluted in mobile phase A (5% acetonitrile, pH 9.8), and separated using a Shimadzu LC-20AB high-performance liquid chromatography (HPLC) system with a Gemini high-pH C18 column (5 μm, 4.6 × 250 mm). The flow rate was 1 mL/min, and the gradient was as follows: 5% mobile phase B (95% acetonitrile, pH 9.8) for 10 min, 5%–35% B for 40 min, 35%–95% B for 1 min, hold at 95% B for 3 min, and re-equilibration in 5% B for 10 min. Elution was monitored at 214 nm, and fractions were collected every 1 min, combined into 10 fractions, and freeze-dried. This procedure was applied for both DDA library construction and DIA quantification.

### Nano-liquid chromatography–tandem MS (nano-LC–MS/MS)

The dried peptide samples were reconstituted in mobile phase A (2% acetonitrile, 0.1% formic acid), centrifuged at 20,000 *g* for 10 min, and analyzed using a Thermo UltiMate 3000 ultra-HPLC system coupled with an Orbitrap Exploris 480 mass spectrometer (Thermo Fisher Scientific, San Jose, CA, US) via a nano-electrospray ionization source. Separation was performed using a self-packed C18 column (150 μm ID, 1.8 μm particles, 35 cm length) at a flow rate of 500 nL/min.

For DDA spectral library construction, the gradient was as follows: 5% B for 0–5 min, 5%–25% B for 5–90 min, 25%–35% B for 90–100 min, 35%–80% B for 100–108 min, 80% B for 108–113 min, and 5% B for 113.5–120 min. MS1 scans were acquired at 350–1650 m/z, at a resolution of 120,000 and with a maximum injection time (MIT) of 90 ms. MS/MS was acquired using Higher-energy Collisional Dissociation (HCD) (Normalized Collision Energy (NCE) 30%) at a resolution of 30,000, dynamic exclusion of 90 s, the top 30 precursors (charge 2 + to 6+, intensity > 2 × 10⁴), and Automatic Gain Control (AGC) targets of 300% for MS and 100% for MS/MS.

For DIA quantification, the gradient was as follows: 5% B for 0–5 min, 5%–25% B for 5–45 min, 25%–35% B for 45–50 min, 35%–80% B for 50–52 min, 80% B for 52–54 min, and 5% B for 54.5–65 min. MS1 scans were acquired at 400–1250 m/z, at a resolution of 120,000 and a MIT of 90 ms. The m/z range was divided into 50 variable isolation windows. MS/MS scans were acquired using HCD (NCE 30%), at a resolution of 30,000 and AGC targets of 300% for MS and 1000% for MS/MS. In SWATH/DIA mode, the entire m/z range was sequentially segmented, and all ions in each segment were fragmented and recorded, allowing comprehensive, unbiased, and reproducible quantification.

### Data processing

The DDA data were processed using the Andromeda search engine in MaxQuant against the UniProt and NCBI RefSeq protein databases to construct the spectral library. The DIA data were analyzed using indexed Retention Time peptides for retention time calibration, and processed using the mProphet algorithm for analytical quality control. A target-decoy approach was used to control the false discovery rate at < 1% at the protein level. Differentially abundance proteins (DAPs) were identified using the MSstats package of R, applying a fold change of > 2 and *P* < 0.05. Protein–protein interaction (PPI) networks were generated using STRING, selecting the top 100 interactions with the highest confidence.

### ELISA analysis for validation

Serum fibrinogen-like protein 2 (FGL2), transforming growth factor beta (TGF-β), and heparanase were measured using commercially available ELISA kits, as follows: FGL2 (LEGEND MAX™ Human sFGL2 ELISA kit, Cat. No. 381558, BioLegend, San Diego, California, USA; intra-assay coefficient of variation [CoV] 5.9%–8.4%, inter-assay CoV 8.7%–10.8%, standard range 0.25–16 ng/mL), TGF-β (Human TGF-β1 Sandwich ELISA kit, Cat. No. KE00002, Proteintech; intra-assay CoV 4.5%–4.9%, inter-assay CoV 2.0%–9.4%, standard range 15.6–1000 pg/mL), and heparanase (Human Heparanase ELISA kit, Cat. No. CSB-E09899h, CUSABIO; intra-assay CoV < 8%, inter-assay CoV < 10%, standard range 0.156–10 ng/mL). All assays were performed in duplicate according to the manufacturers’ instructions. Serum samples were diluted 5-fold for FGL2 and heparanase, and 500-fold for TGF-β.

### Statistical analysis

Continuous data are shown as the mean ± standard deviation, while categorical data are shown as counts. TRAb, TSAb, and duration of graves’ disease and TED are presented as the median and interquartile range. For continuous variables, one-way analysis of variance or the Mann–Whitney U test was used, as appropriate. For log₂ intensity data, the Kruskal–Wallis test was used, followed by Tukey’s or Dunn’s test. These analyses were performed using GraphPad Prism, version 10.4.1 (GraphPad Software, San Diego, CA, USA). To address multicollinearity and prevent overfitting, Least Absolute Shrinkage and Selection Operator (LASSO) logistic regression was performed, with model robustness assessed by 1,000 bootstrap iterations. The area under the receiver operating characteristic (ROC) curve (AUC) and 95% confidence intervals (CIs) were estimated using the percentile method. LASSO was implemented in R 4.5.0 using “glmnet.” Diagnostic accuracy was assessed by ROC curve analysis, with the optimal cutoffs determined according to the Youden index. *P* < 0.05 was considered statistically significant. False discovery rate (FDR) correction using the Benjamini-Hochberg method was applied to the DIA proteomics data. Because the discovery cohort was small relative to the number of quantified proteins and FDR correction was expected to be overly conservative, candidate proteins were selected based on nominal p-values and fold changes, and subsequently validated by ELISA.

## Results

### Participant characteristics

The exploratory DIA proteomics analysis included 16 participants, including 10 patients with TED (six responders and four non-responders), three patients with Graves’ disease, and three healthy controls. The baseline characteristics of participants in the exploratory cohort are summarized in Table 1. In this exploratory cohort, non-responders tended to have a higher proportion of smokers, patients with hyperthyroidism and higher TSAb titer, although these differences did not reach statistical significance. There were no significant differences between responders and non-responders in ophthalmic parameters, such as CAS, diplopia grade, proptosis, or intraocular pressure.

**Table 1:** Background Characteristics of Participants in the Exploratory Proteomics Analysis

Characteristics	TED		*p* value (responder vs. non-responder)	Graves’ disease, *n* = 3	Healthy control, *n* = 3
	Responder, *n* = 6	Non-Responder, *n* = 4			
Age, y	57 (47–64)	43 (18–53)	0.088	40 (36–58)	44 (42–54)
Sex, female/male	4/2	2/2	0.598	3/0	2/1
Smoking habit					
No Yes	5 1	2 2	0.260	2 1	3 0
Dyslipidemia n, (%)	2, (33%)	1, (25%)	0.778		
Thyroid function					
TSH, µIU/mL FT4, ng/dL FT3, pg/mL	0.18 (< LOD-0.57) 1.13 (0.79–1.87) 3.56 (2.80–5.83)	0.05 (< LOD-0.2) 1.54 (1.06–1.70) 4.34 (2.30–10.5)	0.271 0.522 0.831	1.45 (0.98–2.03) 1.34 (1.20–1.53) 3.03 (2.78–3.21)	1.17 (1.02–1.32) 1.01 (0.94–1.19) 3.06 (2.76–3.20)
Euthyroid/ Hyperthyroidism	4/2	1/3	0.197	3/0	3/0
Duration of Graves’ disease, month	12 (2–24)	8 (2–18)	0.539	15 (8–17)	N/A
Antithyroid drug					
n, MMI/PTU/none MMI Dose, mg/day	6/0/0 7.5 (2.5–15)	4/0/0/ 10.0 (5–15)	0.434	3/0/0 5.0(2.5–10)	N/A
TRAb, IU/L	7.5 (1.5–173)	50.5 (4.7–263)	0.456	5.5 (2.9–8.9)	0.7 (0.5–1.4)
TSAb, %	1992 (330–4631)	3422 (2692–4209)	0.394	N/A	N/A
Duration of TED, month	2.5 (2–8)	2.5 (2–4)	0.762	N/A	N/A
CAS	4.5 (3–7)	5.5 (4–6)	0.381	N/A	N/A
Diplopia grade					
Grade 0 Grade 1 Grade 2 Grade 3	0 0 3 3	1 0 2 1	0.392	N/A	N/A
Proptosis				N/A	N/A
Right Left	20.0 (18–26) 19.5 (19–22)	21.0 (12–23) 21.0 (13–27)	0.902 0.617		
IOP					
Right Left	17.0 (12–26) 17.0 (11–23)	17.0 (15–21) 17.0 (15–19)	0.764 1.000	N/A	N/A
Radiation therapy	6, (100%)	3, (75%)	0.197	N/A	N/A

Euthyroid was defined as TSH, FT4, and FT3 all within their reference ranges. Hyperthyroidism was defined as suppressed TSH and/or elevated FT4 or FT3, including subclinical hyperthyroidism (suppressed TSH with normal FT4 and FT3)

N/A: Not available, LOD: Limit of detection, MMI: Methimazole, PTU: Propylthiouracil

Continuous variables are expressed as median (range)

Validation by ELISA was conducted for a total of 59 participants, comprising the 16 patients from the DIA proteomics analysis plus additional participants, including 31 patients with TED (21 responders and 10 non-responders), 14 patients with Graves’ disease, and 14 healthy controls. Comparing the baseline characteristics between responders and non-responders with TED, no significant differences were observed for age and sex. Non-responders were more frequently smokers than responders. A higher proportion of non-responders than responders had hyperthyroidism, including subclinical hyperthyroidism, at the start of TED treatment. The CAS and TSAb titer and its quartile tended to be higher in non-responders, although the difference was not statistically significant. There were no significant differences between responders and non-responders in other clinical parameters, such as diplopia grade, proptosis, or IOP. The clinical characteristics of all 59 participants (21 TED responders, 10 TED non-responders, 14 with Graves’ disease, and 14 healthy controls) are summarized in Table 2.

**Table 2:** Background Characteristics of Participants in the Validation ELISA Analysis

Characteristics	TED		*P*-value Responders vs. Non-Responders	Graves’ disease, *n* = 14	Healthy control, *n* = 14
	Responder, *n* = 21	Non-Responder, *n* = 10			
Age, y	54 (14)	47 (13)	0.228	56 (14)	51 (14)
Sex, female/male	12/9	5/5	0.709	10/4	9/5
Smoking habit			0.034*		
No Yes	18 3	5 5		13 1	13 1
Dyslipidemia n, (%)	5, (24%)	3, (30%)	0.713		
Thyroid function					
TSH, µIU/,L FT4, ng/dL FT3, pg/mL	0.76 (1.37) 1.55 (0.95) 4.01 (1.80)	0.72 (1.09) 1.42 (0.49) 4.64 (2.78)	0.765 0.832 0.807	1.99 (1.01) 1.32 (0.17) 3.43 (0.86)	2.06 (1.90) 1.21(0.23) 3.16 (0.33)
Euthyroid/ Hyperthyroidism	18/3	5/5	0.034*	12/2	14/0
Duration of Graves’ disease, months	4 (2–24)	3 (2–6)	0.662	16 (9–18)	N/A
Antithyroid drug					
n, MMI/PTU/none Dose, mg/day, MMI/PTU	17/1/3 10(5-12.5)/100	7/2/1 7.5(5–10)/200	0.401 0.942	11/2/1 8.6 (5.4)/50	N/A
TRAb, IU/L	18.1 (6.0)	42.2 (18.4)	0.157	2.6 (4.0)	0.8 (0.3)
TSAb, %	792 (1343)	2091 (1943)	0.118	N/A	N/A
TSAb quartile				N/A	N/A
Q1, -489 Q2 490–984 Q3, 985–2597 Q4, 2597-	7, 33% 5, 71% 6, 29% 3, 14%	1, 10% 2, 20% 2, 20% 5, 50%	0.171		
Duration of TED, months	3 (2–5)	2 (2–3)	0.11	N/A	N/A
CAS	4.4 (1.2)	5.2 (1.1)	0.095	N/A	N/A
Diplopia grade			0.516	N/A	N/A
Grade 0 Grade 1 Grade 2 Grade 3	4 3 5 9	1 0 3 6			
Proptosis, mm				N/A	N/A
Right Left	19.3 (3.1) 19.2 (3.5)	21.2 (0.9) 20.9 (1.6)	0.215 0.120		
IOP, mmHg				N/A	N/A
Right Left	16.7(4.7) 16.6 (3.9)	18.8 (3.5) 17.9 (3.8)	0.179 0.364		
Radiotherapy +	20, 95%	9, 90%	0.579	N/A	N/A

Euthyroid was defined as TSH, FT4, and FT3 all within their reference ranges. Hyperthyroidism was defined as suppressed TSH and/or elevated FT4 or FT3, including subclinical hyperthyroidism (suppressed TSH with normal FT4 and FT3)

N/A: Not available

Continuous variables are expressed as mean (SD), except TSAb, TRAb, duration of GD and TED which is expressed as median (IQR)

In both the exploratory and validation cohorts, none of the patients had received thyroidectomy or radioactive iodine therapy for Graves’ disease. The duration of GD and antithyroid treatment status, including the presence, type, and daily dose of antithyroid drugs, are shown in Tables 1 and 2. No apparent differences were observed between TED responders and non-responders.

### Proteomics analysis of the serum based on the DIA analysis

**Fig. 1:**
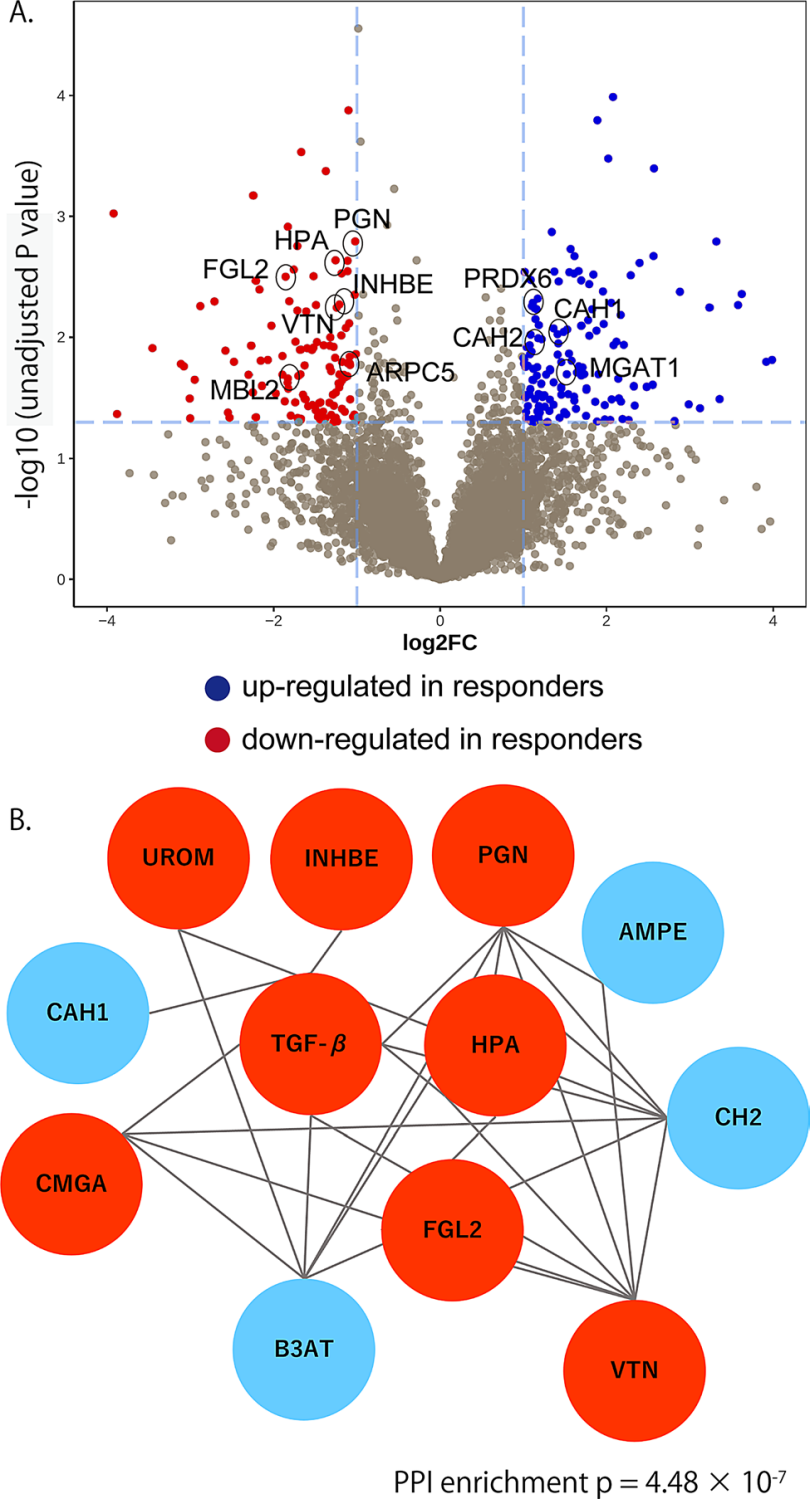
Proteomics analysis comparing responders with non-responders in TED. (**A**) Volcano plot showing differential protein abundance between ivGC responders and non-responders. The y-axis represents -log10 unadjusted p-values. Multiple testing correction was performed using the Benjamini-Hochberg method; however, no proteins remained significant after FDR correction (q < 0.05). Therefore, unadjusted P values are shown for hypothesis-generating visualization. Candidate biomarkers are highlighted with circles. (**B**) PPI network analysis centered on TGF-β. Interactions were analyzed using the STRING database, illustrating the key regulatory nodes and connections among the DAPs. The network showed significant enrichment of protein-protein interactions (PPI enrichment *p* = 4.48 × 10^− 7^). ARPC5, actin-related protein 2/3 complex subunit 5; CAH1, carbonic anhydrase 1; CAH2, carbonic anhydrase 2; CMGA, chromogranin A; DAPs, differentially abundant proteins; DIA, data-independent acquisition; FGL2, fibrinogen-like protein 2; PGN, progranulin; HPA, heparanase; INHBE, inhibin beta E chain; MBL2, mannose-binding lectin 2; MGAT1, alpha-1,3-mannosyl-glycoprotein; PPI, protein–protein interaction; PRDX6, peroxiredoxin 6; TED, thyroid eye disease; TGF-β, transforming growth factor beta; UROM, uromodulin; VTN, vitronectin

Overall, 36,649 peptides and 7,499 proteins were quantified. Among them, 121 proteins were upregulated and 142 proteins were downregulated in non-responders compared with responders. A volcano plot illustrating these differentially abundant proteins (DAPs) is presented in Fig. 1A. PPI network analysis was performed on the 263 DAPs between responders and non-responders. In the PPI analysis, after excluding immunoglobulin fragments, the largest network was composed of 12 DAPs, of which eight were upregulated and four were downregulated in non-responders, centered around TGF-β (Fig. 1B).

The proteomics analysis is registered in the jPOST database (ID JPST003683, PXD061741).

### Candidate predictive biomarker selection

**Fig. 2:**
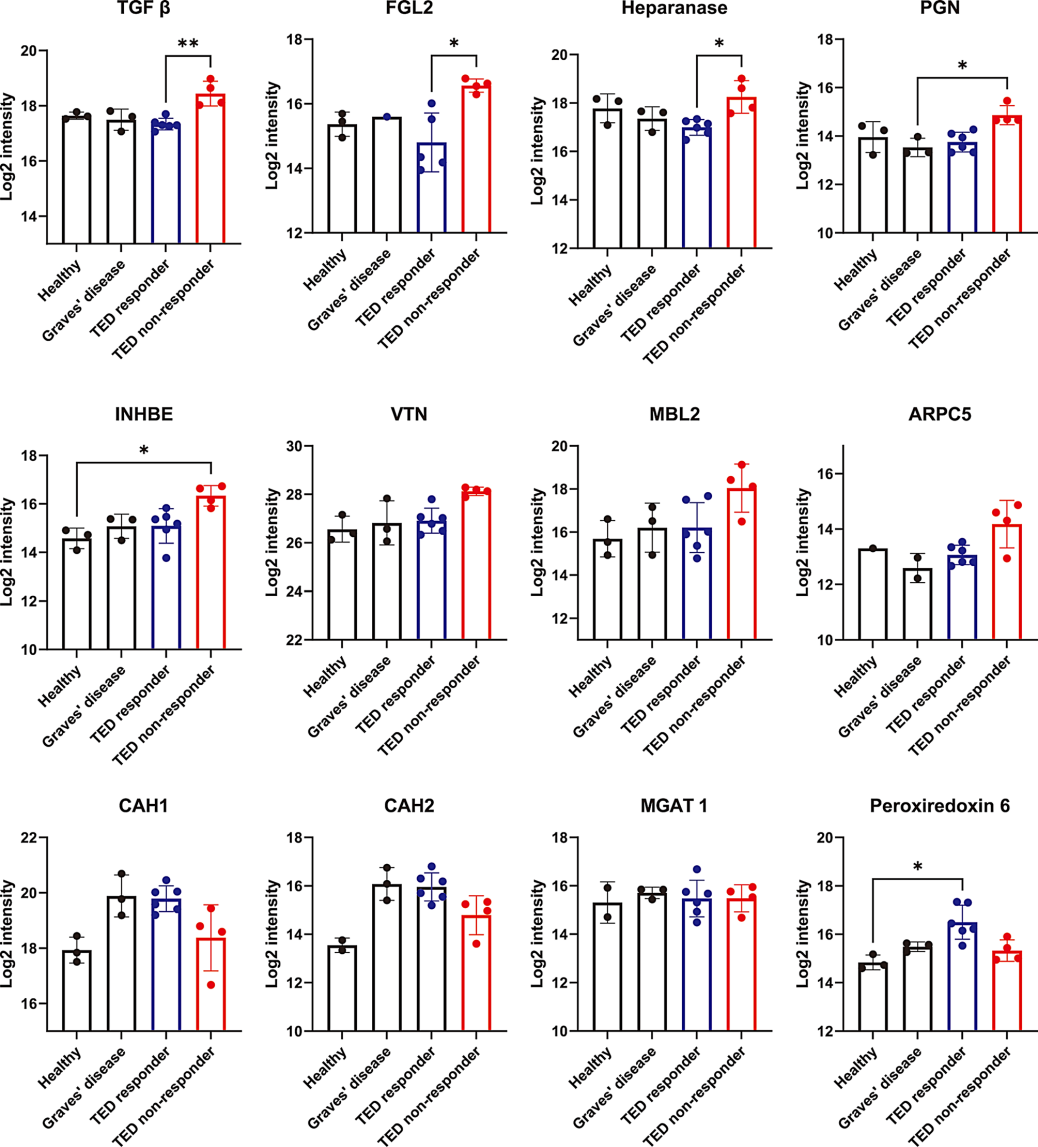
Comparison of protein abundance among the four groups in the DIA proteomics analysis of the 12 candidate biomarker proteins based on the criteria described in the Methods. TGF-β, FGL2, and heparanase, which also showed significant differences between responders and non-responders with TED in the four-group comparison, were selected as final candidate biomarkers. Statistical significance was assessed by the Kruskal–Wallis test. *P < 0.05; **P < 0.01. DIA, data-independent acquisition; FGL2, fibrinogen-like protein 2; TED, thyroid eye disease; TGF-β, transforming growth factor beta

Candidate predictive biomarkers were defined as DAPs between responders and non-responders that (ⅰ) were not immunoglobulin-derived fragments, and (ii) were consistently detected in ≥ 9 of 10 TED discovery samples. We excluded DAPs that were detected in only a few samples because such sparse detection increases susceptibility to outlier-driven effects and reduces the likelihood of reproducible group differences. This initial screening identified 12 protein candidates. To further exclude non-specific DAPs unrelated to TED pathology, the abundance of these 12 proteins was compared across four groups: responders with TED, non-responders with TED, patients with Graves’ disease, and healthy controls. This four-group comparison allowed us to confirm whether the observed differences between responders and non-responders were specific to the TED therapeutic response rather than reflecting differences between disease states or with healthy individuals. When comparing across the four groups, the serum concentrations of TGF-β, FGL2, and heparanase were significantly higher in non-responders than in responders, and they were also higher than in the control groups (Graves’ disease and healthy controls) (Fig. 2). These three proteins were therefore selected as the final candidate predictive biomarkers for validation.

### Validation of candidate predictive biomarkers by ELISA

**Fig. 3:**
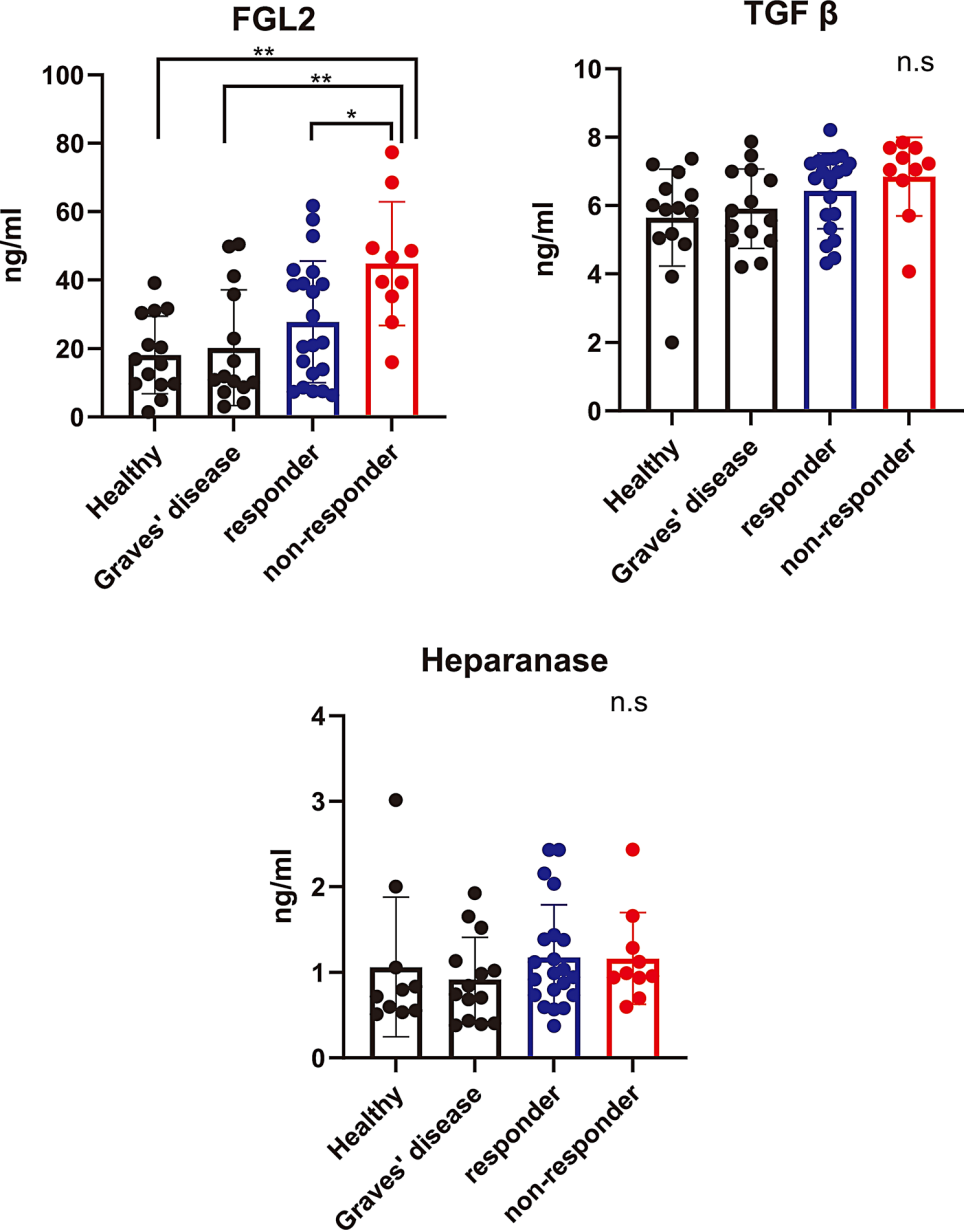
ELISA validation of candidate biomarkers in healthy controls, patients with Graves’ disease, responders with TED, and non-responders with TED. FGL2 was significantly higher in non-responders with TED than in the other groups, but there were no significant differences in TGF-β and heparanase between the groups. One-way analysis of variance followed by Tukey’s post hoc test was used for multiple comparisons. *P < 0.05; **P < 0.01, n.s., not significant. ELISA, enzyme-linked immunosorbent assay; FGL2, fibrinogen-like protein 2; TED, thyroid eye disease; TGF-β, transforming growth factor beta

The 16 samples previously analyzed by DIA proteomics (six responders, four non-responders, three Graves’ disease, and three healthy controls) were subsequently evaluated by ELISA. In the exploratory cohort, serum FGL2 concentrations measured by ELISA showed a moderate correlation with DIA proteomics-derived FGL2 abundance, whereas no significant correlations were observed for TGF-β or heparanase (Supplementary Figure S1). Validation of the three candidate biomarkers was performed using ELISA in a total of 59 serum samples. TGF-β and heparanase were not significantly different among the groups, but FGL2 was significantly higher in non-responders, similar to the DIA analysis (Fig. 3). Serum FGL2 concentrations measured by ELISA in each study group are summarized in Table 3. Because thyroid function (ratio of euthyroid and hyperthyroidism) differed between responders and non-responders, we evaluated whether serum FGL2 levels were influenced by thyroid status across participants of TED and Graves’ disease. No significant difference was observed between euthyroid and hyperthyroid states (Supplementary Figure S2). Thus, the elevation of FGL2 in non-responders is unlikely to be attributable to thyroid dysfunction. Although TSAb and TRAb were higher tendency in non-responders, no significant correlations were observed between serum FGL2 levels and TSAb or TRAb (Supplementary Figure S3).

**Table 3:** Serum FGL2 concentrations measured by ELISA

	*n*	Mean, ng/mL	SD
Healthy	14	17.5	11.2
Graves’ disease	14	21.1	17.2
TED responder	21	27.8	17.8
TED non-responder	10	44.8	18.0

### Predictive accuracy for the therapeutic response

Based on the validation results, FGL2, which was elevated in non-responders, was entered into the multivariate analysis along with clinical factors strongly associated with TED activity, including smoking status, TSAb quartile (Q1–Q4), presence of thyroid dysfunction, age, and female sex. LASSO logistic regression with these six variables identified FGL2 (coefficient = 0.0659), TSAb quartile (coefficient = 0.8199), smoking status (coefficient = 2.2001), age (coefficient = − 0.0109), female sex (coefficient = − 1.1283), and thyroid dysfunction (coefficient = 0.7228) as non-zero contributors in the λ_min model (AUC = 0.86, sensitivity = 0.90, specificity = 0.81, 95% CI 0.65–1.00). Although the per-unit coefficients for FGL2 (per 1 ng/mL) and TSAb (per quartile) were numerically smaller than those for binary predictors, their wide measurement ranges suggested a substantial clinical impact.

**Fig. 4:**
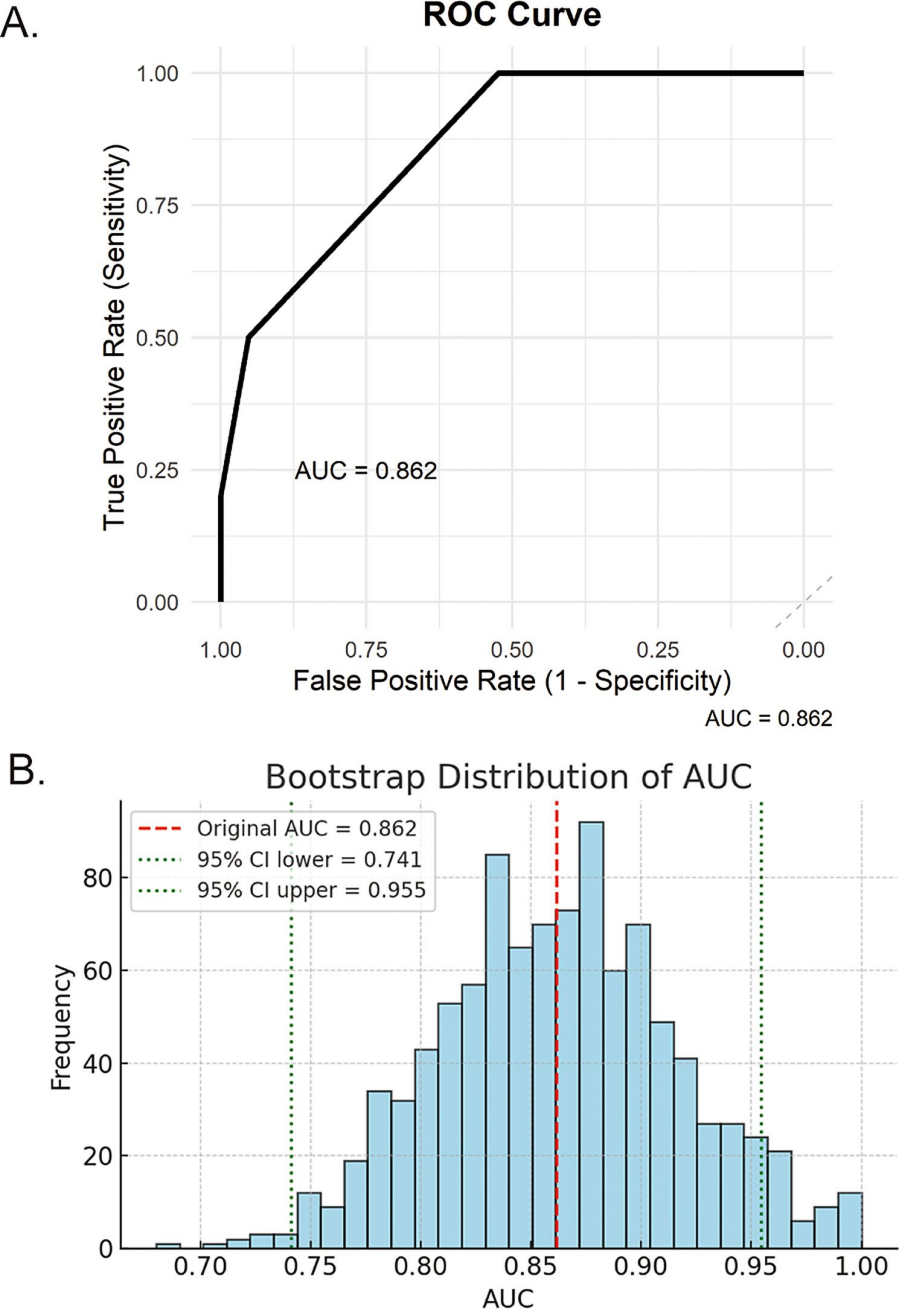
(**A**) ROC curve for the 3-point composite score predicting resistance to ivGC. The composite score was calculated by assigning one point each for serum FGL2 > 39 ng/mL, TSAb > 2597%, and current smoking status, based on predefined cutoff values. The ROC analysis demonstrated good predictive performance of the score for ivGC resistance, with an AUC of 0.86 (95% CI, 0.72–0.95). (**B**) Bootstrap distribution of the AUC based on 1,000 resamples. The red dashed line indicates the original AUC (0.86). The green dotted lines represent the 95% percentile CI (0.74–0.96). TSAb, thyroid-stimulating antibody; AUC, area under the receiver operating characteristic curve; FGL2, fibrinogen-like protein 2; ivGC, intravenous glucocorticoid; ROC, receiver operating characteristic

To develop a prognostic scoring system, smoking, TSAb, and FGL2 were selected as the core predictors. To simplify the scoring model, FGL2 and TSAb were dichotomized based on the optimal cutoff values determined by the ROC curve analysis using the Youden index. In the validation cohort of 31 patients with TED, the optimal cutoff for FGL2 was 39.5 ng/mL (AUC = 0.75, sensitivity = 0.70, specificity = 0.76, 95% CI 0.57–0.93), and for TSAb, the optimal cutoff was the fourth quartile (Q4: >2597%, AUC = 0.71, sensitivity = 0.50, specificity = 0.86, 95% CI 0.52–0.91). ROC analysis for FGL2 and TSAb quartile are shown in Supplementary Figure S4 to illustrate their discriminative performance and cutoff selection. In the final scoring system, 1 point was assigned for each of the following: current smoking, TSAb > Q4, and FGL2 > 39.5 ng/mL. The percentage of responders was 100% (11/11) for patients with a score of 0, 64% (9/14) for those with a score of 1, 25% (1/4) for those with a score of 2, and 0% (0/2) for those with a score of 3. The scoring system demonstrated high predictive performance, with an optimal cutoff of ≥ 1 point (AUC = 0.86, sensitivity = 1.0, specificity = 0.52, 95% CI 0.72–0.95; Fig. 4A), whereas the exclusion of FGL2 from the scoring system reduced the accuracy (AUC = 0.78, sensitivity = 0.80, specificity = 0.72, 95% CI 0.62–0.95).

Internal validity was assessed using bootstrap resampling (1,000 iterations). The original AUC was 0.86 (95% CI 0.74–0.96) (Fig. 4B), indicating robust discriminative performance across the resampled datasets.

## Discussion

In the present study, DIA proteomics analysis of the serum was applied to identify candidate predictive biomarkers of the therapeutic response to ivGC therapy in patients with TED. Based on the DIA analysis, FGL2, TGF-β, and heparanase were identified as candidate predictive biomarkers. ELISA validation of these biomarker candidates in a larger patient cohort showed that FGL2 was the biomarker that was most consistently associated with the treatment response. When FGL2 was combined with TSAb quartile and smoking status, which is implicated in the pathogenesis of TED, the accuracy of predicting a therapeutic response to ivGC therapy was improved.

FGL2 has been implicated in several autoimmune diseases. Serum FGL2 is primarily secreted by regulatory T cells (Tregs) [14] and plays an immunosuppressive role by inhibiting dendritic cell maturation, inducing B-cell apoptosis, inhibiting M1 macrophage polarization, and selectively inhibiting the differentiation of T helper (Th)1 and Th17 cells [15]. FGL2 has been reported to suppress acute rejection of liver transplants, food allergies, and lupus-like autoimmunity [16]. Recombinant FGL2 is also expected to be a novel therapeutic agent for lupus-like autoimmunity, glomerulonephritis, and multiple sclerosis [16]. In the present study, FGL2 was not associated with the presence of Graves’ disease, hyperthyroidism, or TED clinical activity and severity. Rather, it appeared to be associated with the responsiveness of patients with TED to ivGC therapy. In inflammatory bowel disease, Tregs upregulate FGL2 secretion to suppress highly activated effector T cells. However, this is insufficient to halt the excessive inflammatory response, resulting in persistent inflammation. Against this background, elevated FGL2 has been demonstrated to be a useful biomarker of disease activity in inflammatory bowel disease [17]. In ivGC-resistant TED, we observed increased FGL2, suggesting that FGL2 may indirectly reflect orbital inflammation that cannot be fully suppressed by ivGC therapy.

Although several studies have investigated the efficacy of steroid therapy in the context of TED [18–20], a particularly notable study investigating the mechanisms of resistance reported that Th17.1 expression is markedly increased in severe steroid-resistant TED [21]. Th17.1 is a type of Th cell with both Th1 and Th17 characteristics that produces interferon-γ and interleukin-17 A [22]. In orbital tissues from severe steroid-resistant TED (compared with steroid-responsive TED), infiltration of Th17.1 cells persisted even after steroid therapy, which was accompanied by markedly elevated interferon-γ expression, which has been reported as a major factor contributing to steroid resistance. FGL2 has been shown to counteract interferon-γ [23] and interleukin-17A [24] secretion by Th17.1 cells. We hypothesize that the FGL2 elevation in patients with ivGC-resistant TED observed in this study may have resulted from enhanced Th17.1 expression, leading to increased interferon-γ and interleukin-17 A secretion, in turn upregulating FGL2 to counteract these pro-inflammatory cytokines. FGL2 upregulation may serve as a surrogate marker of excessive Th17.1 cell activation. When FGL2 exceeds a certain threshold, Th17.1 activity may no longer be effectively controlled by ivGC therapy, resulting in persistent orbital interferon-γ overexpression and ultimately leading to irreversible tissue destruction. 

In this study, combining FGL2, TSAb quartile, and smoking status improved treatment response prediction. TSAb plays a central role in humoralimmunity in TED and correlates with severity [25, 26]. Moreover, high TSAb is linked to a poor ivGC response [27]. Smoking contributes to TED pathogenesis via oxidative stress, hypoxia, and orbital fibroblast activation, promoting fibrosis [28] and impairing ivGC efficacy [29]. However, TSAb and smoking alone insufficiently predict resistance. With respect to TSAb, accumulating evidence suggests that dynamic changes during treatment may be as important as, or even more informative than, baseline pretreatment levels in predicting therapeutic response [27]. Consistent with this notion, in the present study, a substantial number of patients with elevated pretreatment TSAb levels nevertheless achieved a favorable response to ivGC. Importantly, no significant correlation was observed between serum FGL2 concentrations and TSAb levels, indicating that FGL2 captures a distinct biological aspect of the disease. Accordingly, integrating FGL2 with conventional risk factors provided complementary information and enhanced predictive performance. In our scoring system, scores of 1 and 2 encompassed both responders and non-responders, limiting the prediction ability. Notably, extreme scores (0 or 3) predicted responsiveness or resistance with high accuracy. These extremes accounted for 42% of the patients (13/31), indicating that nearly half of the patients could be classified with high certainty. These findings suggest that integrating FGL2 with conventional factors may markedly improve ivGC treatment response prediction. Patients with high predictive scores for FGL2, TSAb, and smoking may benefit from alternative first-line treatments instead of ivGC therapy. Many studies have sought to identify reliable predictors of responsiveness to glucocorticoid therapy in patients with active TED. Treatment response has been evaluated using clinical background factors such as age and baseline CAS [30], as well as blood-based biomarkers including cytokines [19] and microRNAs such as miR-146a [20]. In addition, predictive nomograms incorporating detailed ophthalmologic parameters, such as meibomian gland length and superficial retinal vascular density, have been reported [31]. Associations between pretreatment selenium status and glucocorticoid responsiveness have also been described [32]. Although several promising predictors have been proposed, studies exploring novel biomarkers are often limited by small sample sizes, underscoring the need for large-scale validation. Similarly, while FGL2 identified in the present study requires further validation in larger cohorts, it may represent a potential candidate as a novel biomarker for predicting glucocorticoid responsiveness in TED. 

### Limitations

This study has several limitations. First, although FDR correction was applied to the DIA proteomics data, no proteins remained significant after adjustment. Given the small discovery cohort relative to the number of quantified proteins, the DIA analysis was regarded as hypothesis-generating, and validation by ELISA was prioritized to enhance reliability. Second, the number of ivGC-resistant patients was limited, resulting in a low event-to-predictor ratio for multivariate modeling. Although LASSO regularization and bootstrap resampling were used to mitigate overfitting, the stability of the estimates may still be affected. Third, treatment regimens in this cohort reflect real-world practice in Japan and differ from EUGOGO-recommended protocols; therefore, generalizability to other therapeutic settings requires further evaluation. Fourth, potential confounding factors related to antithyroid treatment and metabolic status should be considered. Because the vast majority of patients were treated with methimazole, with only a small number receiving propylthiouracil, we were unable to meaningfully assess the impact of different antithyroid drug types on serum FGL2 concentrations. Other potential factors associated with FGL2, such as selenium status and the gut microbiome, were not evaluated in this study and warrant further investigation. Fifth, treatment response was defined solely by CAS improvement, and broader endpoints such as the EUGOGO Composite Index or imaging parameters may provide more comprehensive assessments. Finally, because ivGC-requiring TED is rare, the exploratory DIA samples were necessarily included in the ELISA validation cohort. Independent confirmation in a larger, non-overlapping cohort will be essential to verify the reproducibility and clinical applicability of FGL2 as a predictive biomarker.

## Conclusion

By applying DIA-based proteomics analysis followed by ELISA validation, we identified serum FGL2 as a novel biomarker associated with the treatment response to ivGC therapy in patients with active TED. To our knowledge, this is the first study to evaluate FGL2 as a pre-treatment predictor of ivGC efficacy. Incorporating FGL2 together with TSAb and smoking status provides a promising scoring system to distinguish ivGC-resistant patients with TED, which may support personalized treatment selection in the evolving landscape of TED management.

## Supplementary Information

Below is the link to the electronic supplementary material.

Supplementary Material 1: Supplementary Figure S1: Correlations between serum protein abundance quantified by DIA proteomics and serum concentrations measured by ELISA in the exploratory cohort. Associations were assessed using Spearman’s rank correlation analysis. A significant positive correlation was observed for FGL2 (ρ = 0.59, *P* = 0.036), whereas no significant correlations were detected for TGF (ρ = 0.019, *P* = 0.47) or HPA (ρ = 0.04, *P* = 0.89). DIA, data-independent acquisition; ELISA, enzyme-linked immunosorbent assay; FGL2, fibrinogen-like protein 2; TGF-β, transforming growth factor beta; HPA, heparanase.

Supplementary Material 2: Supplementary Figure S2: Serum FGL2 levels stratified by thyroid function (Euthyroid vs. hyperthyroid) among patients with TED and Graves’ disease. Bar graphs represent group means with standard deviations, and individual data points are overlaid to illustrate sample distribution. No significant difference was observed between euthyroid and hyperthyroid groups (Mann-Whitney U-test, *p* = 0.626), indicating that thyroid dysfunction alone dose not account for the elevated FGL2 levels seen in ivGC-resistant patients.

Supplementary Material 3: Supplementary Figure S3: Correlations between serum FGL2 levels and thyroid-stimulating antibody (TSAb) or thyrotropin receptor antibody (TRAb). Associations were assessed using Spearman’s rank correlation analysis. Spearman’s correlation coefficients (ρ) and corresponding P value are shown.

Supplementary Material 4: Supplementary Figure S4: Receiver operating characteristic (ROC) curve analyses for serum FGL2 and TSAb quartiles in discriminating responders from non-responders to ivGC. The area under the curve (AUC), 95% confidence intervals, and P values are shown. Cutoff values were determined using the Youden index.

Supplementary Material 5: Upregulated proteins in responders compared with non-responders identified by DIA proteomic analysis.

Supplementary Material 6: Downregulated proteins in responders compared with non-responders identified by DIA proteomic analysis.

## Data Availability

The proteomics data generated in this study have been deposited in the jPOST Repository and are publicly available under the accession numbers JPST003683 and PXD061741. The dataset can be accessed at https://repository.jpostdb.org/entry/JPST003683.
