# Supplementary figures and images for: A preliminary study developing a scoring model incorporating fibrinogen-like protein 2 for predicting glucocorticoid resistance in thyroid eye disease
Source: Thyroid Res. 2026 Apr 1;19:13. doi: 10.1186/s13044-026-00293-8 (PMC13040981; doi:10.1186/s13044-026-00293-8)

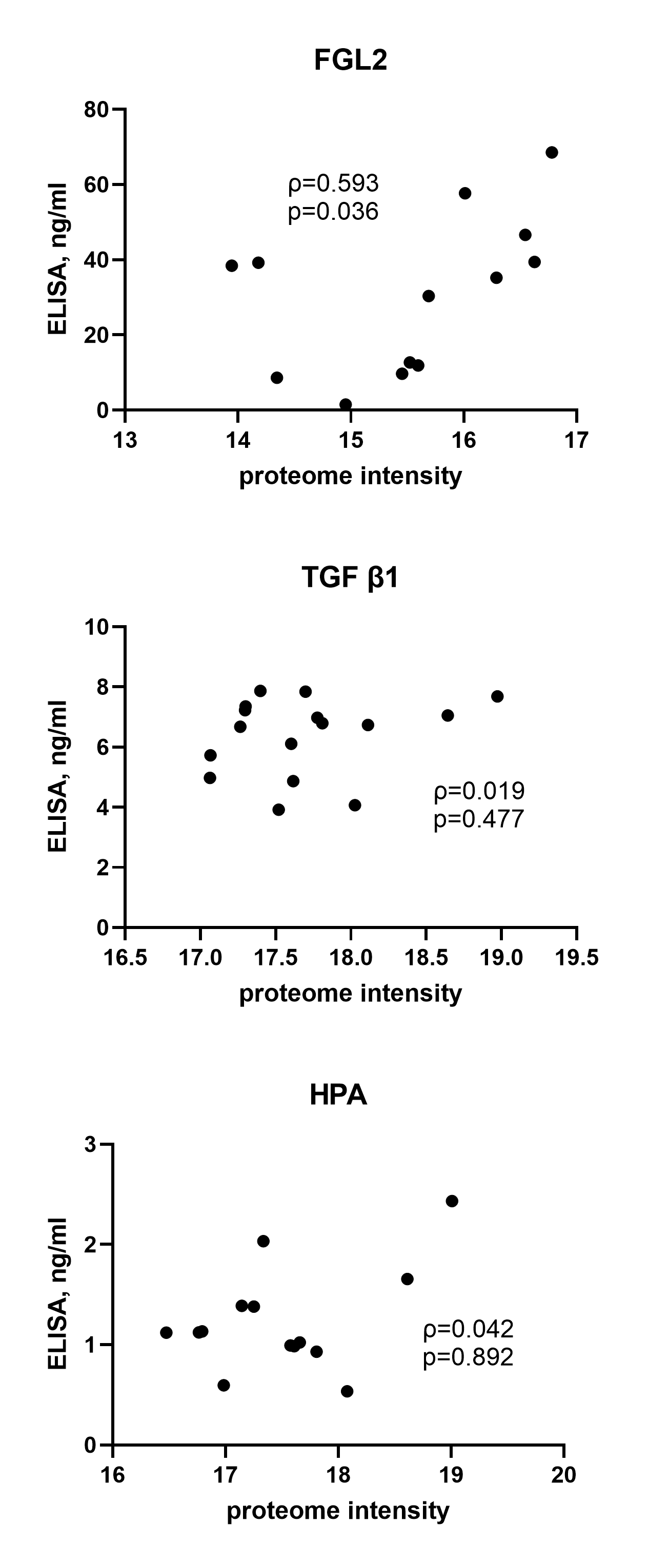

Supplement: Supplementary file 1 — Supplementary Material 1: Supplementary Figure S1: Correlations between serum protein abundance quantified by DIA proteomics and serum concentrations measured by ELISA in the exploratory cohort. Associations were assessed using Spearman’s rank correlation analysis. A significant positive correlation was observed for FGL2 (ρ = 0.59, P = 0.036), whereas no significant correlations were detected for TGF (ρ = 0.019, P = 0.47) or HPA (ρ = 0.04, P = 0.89). DIA, data-independent acquisition; ELISA, enzyme-linked immunosorbent assay; FGL2, fibrinogen-like protein 2; TGF-β, transforming growth factor beta; HPA, heparanase. [file 13044_2026_293_MOESM1_ESM.tif]

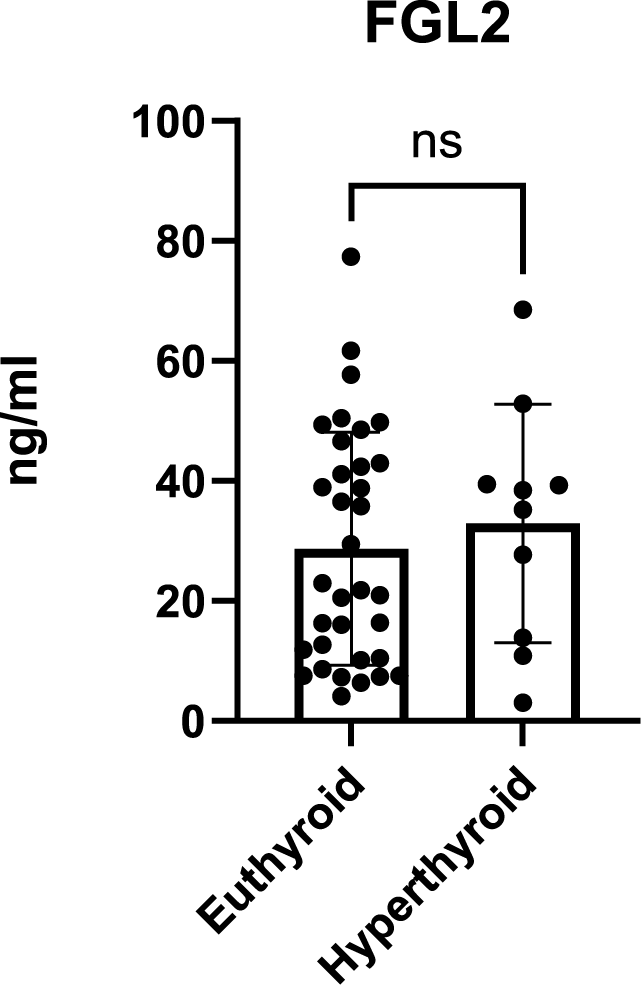

Supplement: Supplementary file 2 — Supplementary Material 2: Supplementary Figure S2: Serum FGL2 levels stratified by thyroid function (Euthyroid vs. hyperthyroid) among patients with TED and Graves’ disease. Bar graphs represent group means with standard deviations, and individual data points are overlaid to illustrate sample distribution. No significant difference was observed between euthyroid and hyperthyroid groups (Mann-Whitney U-test, p = 0.626), indicating that thyroid dysfunction alone dose not account for the elevated FGL2 levels seen in ivGC-resistant patients. [file 13044_2026_293_MOESM2_ESM.tif]

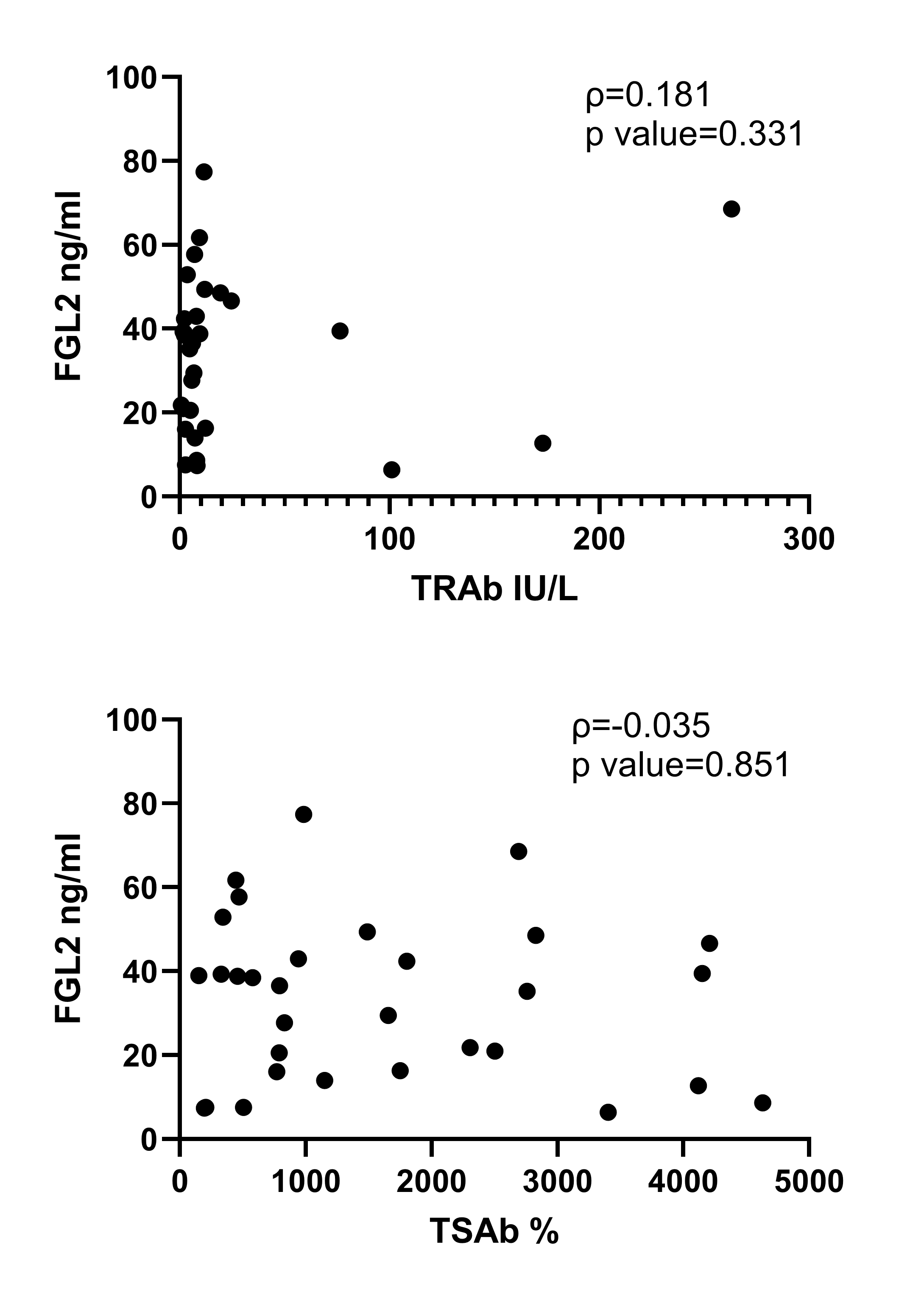

Supplement: Supplementary file 3 — Supplementary Material 3: Supplementary Figure S3: Correlations between serum FGL2 levels and thyroid-stimulating antibody (TSAb) or thyrotropin receptor antibody (TRAb). Associations were assessed using Spearman’s rank correlation analysis. Spearman’s correlation coefficients (ρ) and corresponding P value are shown. [file 13044_2026_293_MOESM3_ESM.tif]

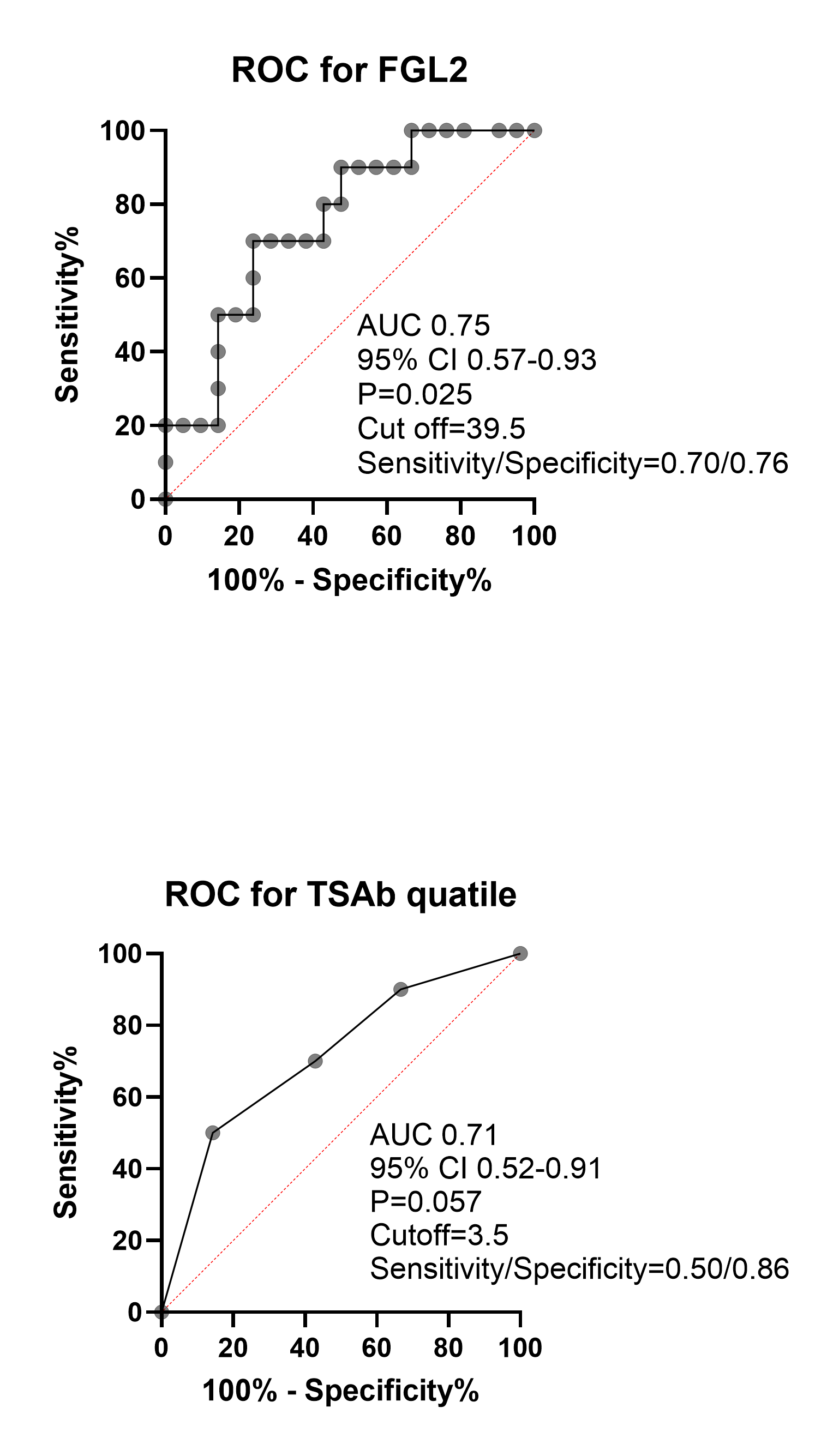

Supplement: Supplementary file 4 — Supplementary Material 4: Supplementary Figure S4: Receiver operating characteristic (ROC) curve analyses for serum FGL2 and TSAb quartiles in discriminating responders from non-responders to ivGC. The area under the curve (AUC), 95% confidence intervals, and P values are shown. Cutoff values were determined using the Youden index. [file 13044_2026_293_MOESM4_ESM.tif]
